# Patch-clamp studies and cell viability assays suggest a distinct site for viroporin inhibitors on the E protein of SARS-CoV-2

**DOI:** 10.1186/s12985-023-02095-y

**Published:** 2023-07-08

**Authors:** Ulrike Breitinger, Christine Adel Sedky, Heinrich Sticht, Hans-Georg Breitinger

**Affiliations:** 1 Department of Biochemistry, German University in Cairo, Main Entrance of Al Tagamoa Al Khames, New Cairo, 11835 Egypt; 2 Division of Bioinformatics, Institute for Biochemistry, Friedrich-Alexander-Universität Erlangen-Nürnberg, Erlangen, Germany

**Keywords:** SARS-CoV-2, E protein, Viroporins, Cell viability assay, Patch-clamp electrophysiology, Viroporin inhibitors, Ivermectin derivatives

## Abstract

**Background:**

SARS-CoV-2 has caused a worldwide pandemic since December 2019 and the search for pharmaceutical targets against COVID-19 remains an important challenge. Here, we studied the envelope protein E of SARS-CoV and SARS-CoV-2, a highly conserved 75–76 amino acid viroporin that is crucial for virus assembly and release. E protein channels were recombinantly expressed in HEK293 cells, a membrane-directing signal peptide ensured transfer to the plasma membrane.

**Methods:**

Viroporin channel activity of both E proteins was investigated using patch-clamp electrophysiology in combination with a cell viability assay. We verified inhibition by classical viroporin inhibitors amantadine, rimantadine and 5-(N,N-hexamethylene)-amiloride, and tested four ivermectin derivatives.

**Results:**

Classical inhibitors showed potent activity in patch-clamp recordings and viability assays. In contrast, ivermectin and milbemycin inhibited the E channel in patch-clamp recordings but displayed only moderate activity on the E protein in the cell viability assay, which is also sensitive to general cytotoxic activity of the tested compounds. Nemadectin and ivermectin aglycon were inactive. All ivermectin derivatives were cytotoxic at concentrations > 5 µM, i.e. below the level required for E protein inhibition.

**Conclusions:**

This study demonstrates direct inhibition of the SARS-CoV-2 E protein by classical viroporin inhibitors. Ivermectin and milbemycin inhibit the E protein channel but their cytotoxicity argues against clinical application.

## Background

Coronaviruses have been responsible for major outbreaks of respiratory diseases SARS, MERS, and the 2019 epidemic of COVID-19, caused by SARS-CoV-2 [1]. Severe infection with SARS-CoV-2 results in severely impaired lung function, often associated with systemic inflammation and a massive release of inflammatory cytokines, known as cytokine storm [2–4], which is also known in other virus-related diseases [5–7]. A worldwide vaccination campaign was essential to reduce the spread of COVID-19, yet new strains of SARS-CoV-2 have emerged, and there is a persistent risk of the appearance of new strains that are partially [8], or even completely resistant to current vaccines. In addition to prevention of infection through vaccination, effective treatment of infected patients is an essential need in the fight against SARS-CoV-2.

SARS-CoV-2 is an enveloped, single-stranded positive sense RNA virus comprising 14 open reading frames in its genome. These encode for structural proteins, forming the virus capsid, including the spike protein (S), membrane protein (M), envelope protein (E), and the nucleocapsid protein (N), as well as non-structural proteins, including those of the viral replicase and protease apparatus, and accessory proteins [3].

The envelope protein E, and the ORF3a protein of SARS-CoV-2 belong among the class of viroporins, a group of mostly small, hydrophobic integral membrane proteins that assemble into membrane channels, usually located in intracellular membranes of ER and Golgi apparatus [9–11]. Being essential for virus replication and release, viroporins are indeed viable targets for antiviral drugs [9, 10, 12, 13]. The first well-characterized viroporin was the M2 channel of influenza A Virus [14–16]. Other viroporins are the p7 channel of Hepatitis C [17, 18] and the Viral protein U of human immunodeficiency virus [19]. Viroporins are involved in the viral infection cycle in two ways, (i) causing ionic imbalances and disrupting pH gradients through their action as intracellular ion channels, and (ii) by disrupting cellular pathways through protein–protein interactions [2, 9, 12, 20].

E proteins are found in all coronaviruses, and despite variation in protein size (75–109 amino acids) and sequence [21], their structure is highly conserved, including a short N-terminal segment, a long alpha helical section likely including one transmembrane domain and an unstructured carboxy terminus [9]. The sequence of the E protein is highly conserved between SARS-CoV (1-E) and SARS-CoV-2 (2-E), which differ by one deletion and three exchanged residues, all located near the C-terminal end of the 76-amino acid protein.

Studies on recombinantly expressed SARS-CoV E protein identify the protein at the cell plasma membrane [22], yet this could not be confirmed in later studies [23]. Viroporin channel activity was confirmed using planar lipid bilayers and micelles where the E protein forms oligomeric structures that build the ion channel pore [22, 24, 25].

The genome of SARS-CoV encodes three putative viroporins, the E protein, ORF3a, and ORF8a proteins [12]. Activity of E and 3a proteins is required for maximal SARS-CoV replication and virulence, with reduced viral viability if one of the two viroporins was inactive, and complete loss of viral replication if both were absent. In contrast, the ORF 8a protein was not required for virus activity [12]. The same study showed in a mouse model of SARS-CoV infection that both, ion channel activity of the E protein, as well as intracellular interactions mediated by its PDZ-binding motif are necessary for virulence [12]. Deletion of the E gene in different coronaviruses leads to reduced virus maturation and release, production of low-virulence virus, and a reduction of cellular stress and virus-induced apoptosis [26, 27]. Both, E- as well as ORF3a protein of SARS-CoV and SARS-CoV-2 show high sequence conservation and similar function, while ORF8 proteins were not reported to be viroporins [9]. Thus both, E and ORF3a are viable pharmaceutical targets [12, 26], and an inhibitor, or a combination of inhibitors that is able to target both viroporins would be a promising therapeutic tool for the treatment of COVID-19.

Classical viroporin inhibitors include amantadine and rimantadine [18, 28, 29], 5-(N,N-hexamethylene)amiloride (HMA) [22, 30], and several iminosugar derivatives [29, 31]. Recently, we have shown that some members of the flavonoid family are inhibitors of recombinant SARS-CoV E protein channels [32].

Ivermectin, a well-characterized anthelminthic and ion channel modulator [33, 34] has been explored in numerous studies for its potential use in the fight against SARS-CoV-2 [35], but results are ambiguous, with some activity observed only in some states of infection [36–39], and ivermectin-associated toxicity limiting clinical use, similar to its action against Newcastle virus [36]. Here, we tested ivermectin and three of its derivatives, namely nemadectin, milbemycin and ivermectin aglycon for their activity against recombinant E protein of SARS-CoV-2 expressed in HEK293 cells. In cell viability studies, the effect of ivermectin on E protein activity was noted but not separable from the cytotoxicity of ivermectin itself. In patch-clamp electrophysiological studies, ivermectin was a potent inhibitor of the E protein, milbemycin also showed activity, while inhibition by ivermectin aglycon was less pronounced, and nemadectin was inactive. Thus, we identify the E protein of SARS-CoV-2 as a direct pharmacological target for viroporin inhibitors. While some ivermectin derivatives were confirmed to be potent inhibitors of the E protein channel, clinical applicability of these compounds is limited by their cytotoxicity.

## Methods

*Generation of the SARS-CoV-2 E protein construct* cDNA encoding the E protein of SARS-CoV (1-E) [32] was used as template to introduce the required mutations (T55S, V56F, E69R, G70del) to generate a plasmid encoding the SARS-CoV-2 E protein (2-E) using site-directed mutagenesis through an overlap extension PCR protocol [18]. 2-E cDNA was inserted into the pRK5 vector using EcoRI and PstI restriction sites. For electrophysiological measurements, a membrane-directing signal peptide ‘MWTPRVPPPRPALSFFLLLLLGVTYGLFPEEPPPLSVAE’ from murine semaphorin-6B, followed by a myc-tag for Western blot analysis were fused to the N-terminus of the E protein. All constructs were verified by sequencing. E protein was expressed in HEK293 cells for Western blot analysis, MTT assays and electrophysiological measurements.

*Cell culture and transfection* HEK293 cells (ATCC, LGC Standards GmbH, Wesel, Germany) were cultured in 10 cm tissue culture dishes in Dulbecco's Modified Eagle Medium (DMEM, Sigma-Aldrich, Cairo, Egypt) supplemented with 10% FBS (Invitrogen, Karlsruhe, Germany) and 1000 IU of Penicillin/Streptomycin (Sigma-Aldrich, Cairo, Egypt) at 5% CO_2_ and 37 °C in a water-saturated atmosphere.

*Western blot analysis* Cells were plated in 6 cm plates and transfected using 4 µg of cDNA and 12 µg of PEI per plate. Three days after transfection, HEK293 cells were harvested, a crude membrane fraction prepared by two rounds of homogenization followed by centrifugation at 14000 g for 20 min, and subjected to SDS-PAGE and Western blotting. A rabbit polyclonal anti-c-myc primary antibody (Proteintech, Martinsried, Germany), and an alkaline phosphatase-conjugated goat anti rabbit secondary antibody (Proteintech, Martinsried, Germany) were used and the blot visualized using 0.03% nitro blue tetrazolium and 0.02% 5-bromo-4-chloro-3-indolyl-phosphate in substrate buffer (100 mM Tris-HCl, pH 9.5; 100 mM NaCl; 5 mM MgCl_2_). Since the E-protein was unstable and degraded quickly, all operations were performed immediately after harvest, using phosphate-buffered saline containing protease inhibitors (Roche C@mplete, Sigma-Aldrich, Deisenhofen, Germany), and heated to 95 °C for 2 min immediately prior to loading of the SDS gel. This minimized degradation, however, oligomers are not fully separated upon this treatment. Protein concentration was determined using a Qbit Assay (Thermofisher, Karlsruhe, Germany), and 20 μg of total protein were applied per lane.

**Fig. 1:**
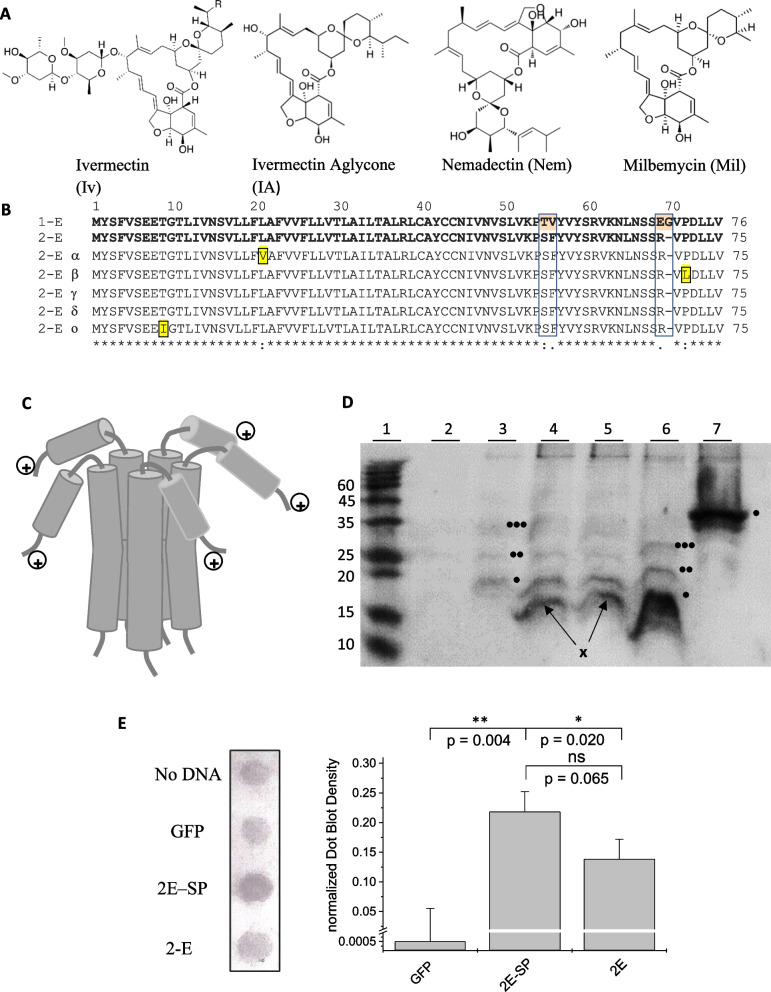
**A** Ivermectin derivatives used in this study. **B** Sequence alignment of E proteins from SARS-CoV (1-E), SARS-CoV-2 (2-E), and recent 2-E variants. Boxes indicate the four positions of difference between 1-E (orange) and 2-E, yellow colour indicates amino acid exchanges found in 2-E of new SARS-CoV-2 variants. **C** Side and top view structural model of the CoV E protein (PDB code: 5 × 29) determined by NMR spectroscopy in lyso-myristoyl phosphatidyl-glycerol micelles [59] The five subunits are colour coded; the N- and C-terminal residues are labelled for one subunit only. **D** Western blot analysis of 2-E protein and control viroporins expressed in HEK293 cells. All viroporins had an N-terminal myc-tag. Prior to loading, samples were heated briefly to 95 °C to minimize degradation, but multimers were not completely separated. Staining was performed using a primary anti-myc antibody and an AP-conjugated secondary AB. Lane 1: Ladder (ROTI^®^Mark TRICOLOR: 10, 15, 20, 25, 35, 45, 60, 75); lane 2: GFP; lane 3: SARS-CoV-2 E protein plus signal peptide; lane 4: SARS-CoV-2 E protein without signal peptide; lane 5: SARS-CoV E protein without signal peptide; lane 6: hepatitis C virus p7-1a; lane 7: SARS-CoV-2 ORF3a. Expected molecular weights (kD): SARS-CoV-2 E: 12.6; SARS-CoV-2 E + SP: 16.8, Hepatitis C virus p7-1a: 11.3; SARS-CoV-2 ORF 3a: 35.6 kD Signal peptide (SP): 4.2; SARS-CoV-2 E dimer: 25.2; trimer: 37.8 kD; Hepatitis C virus p7-1a dimer: 22.6; trimer: 33.9. Note immune signal is only observed for myc-tagged antibodies. Small viroporins do not migrate in the same way as globular marker proteins. Suggested oligomers (●, ●●, ●●●) and degradation bands (x) are indicated between lanes 3 and 4 for E proteins, and right of lane 6 and 7 for p7-1a and ORF3a, respectively. **E** Surface Expression Analysis: HEK293 cells were transfected with cDNA encoding E protein (E), and E protein with membrane-directing signal peptide (E–SP), untransfected and mock-transfected cells were used as control. Left panel: Dot blot. Right Panel: Quantification of Blot intensities, average ± standard deviation from three independent experiments with two spots per treatment (n = 6) is shown. The y-axis break is between 0.001 and 0.02. Background signal (untransfected cells = No DNA) was subtracted from spot intensities; p-values are indicated, **: p < 0.01, ns: not significant, p > 0.05 (one-way ANOVA)

*Cell surface expression assay* HEK293 cells were transfected with cDNA constructs encoding myc-tagged SARS-CoV-2 E protein (2-E), and SARS-CoV-2 E protein with membrane-directing signal peptide (2E–SP). Untransfected HEK293 cells, and cells transfected with GFP were used as control. Live cells were incubated with a rabbit primary anti-myc antibody for 1 h, then with a secondary alkaline phosphatase-coupled goat-anti-rabbit antibody for 30 min. Cells were then harvested, homogenized, and the cell suspension spotted onto wells of a Dot blot apparatus. The blot was developed using NBT/BCIP and analysed. Pixel intensity was quantified using ImageJ software without further correction of the raw data. Pixel intensities for each peak were normalized to the total pixel intensity of all peaks. Averages were taken of duplicate readings from three independent expression experiments (n = 6). For display (Fig. 1E), background intensity (untransfected cells) was subtracted from the recorded spot intensities.

*Cell viability assay* cell viability assays were performed as described before [29]. Briefly, HEK293 cell suspension was seeded into 96-well plates and transfected one day after plating using 0.3 µg DNA and 0.6 µg of PEI per well. Stock inhibitor (5 µl end volume) in different concentrations was added and MTT (3-(4,5-dimethylthazolk-2-yl)-2,5-diphenyl tetrazolium bromide) assay was performed after three days as described [29]. Viable cells produce a purple-coloured formazan derivative of MTT which was dissolved by careful removal of MTT solution and addition of 100 µl of DMSO per well. Absorbances at 595 nm were recorded using a Victor-3 plate reader (Perkin-Elmer, Berlin, Germany). Viability of cells transfected with 2-E expression construct were normalized to control cells transfected with empty pRK5 vector. Background absorbance (200 mM KCl) was subtracted. Next, we divided test readings by control readings (mock-transfected cells), thereby normalizing control data to the value 1. We compared E protein from SARS-CoV and SARS-CoV-2 under identical experimental conditions. Additional controls included: (1) GFP transfected cells to verify efficient transfection; (2) comparison of untransfected cells to pRK5 transfected cells to identify the effect of transfection on cell viability; (3) addition of 200 mM KCl to induce complete cell death. (4) To test whether the inhibitors had any effect on cell viability, they were also added to mock (empty vector) transfected cells. Cell viability of inhibitor-treated cells transfected with E protein was normalized to the viability of control cells treated with the same concentration of inhibitor.

*Electrophysiological recordings and data analysis* Cells were plated on acetone treated glass coverslips in 24 well plates, transfected one day after passage using 1 µg of E protein cDNA, 1 µg of green fluorescence protein (GFP) cDNA and 3 µl polyethyleneimine (PEI) (1 mg/ml) (Sigma-Aldrich, Cairo, Egypt) per well. Patch-clamp recordings were done 2–3 days after transfection. For recording, cells were kept in a bathing solution (external buffer) containing 135 mM NaCl, 5.5 mM KCl, 2 mM CaCl_2_, 1.0 mM MgCl_2_, and 10 mM Hepes (pH 7.4 with NaOH). Recording pipettes were pulled from borosilicate glass (World Precision Instruments, Berlin, Germany) using a Sutter P-97 horizontal puller (Sutter, Novato, CA). The intracellular buffer was (in mM) 140 CsCl, 1.0 CaCl_2_, 2.0 MgCl_2_, 5.0 EGTA and 10 Hepes (pH adjusted to 7.2 with CsOH). Control cells were transfected with pRK5 vector co-transfected with GFP and compared to cells transfected with E protein and GFP. E protein channel activity was assessed using current–voltage relations from a voltage ramp ranging from − 60 mV to + 50 mV in 10 mV steps. For measurements of inhibition, test compounds were added to the extracellular bath. A minimum of 6 cells per condition were recorded and averaged.

## Results

To compare the function and inhibition of E proteins from SARS-CoV (1-E) and SARS-CoV-2 (2-E), we generated 2-E constructs by introducing exchange/removal of the four positions that are different between 2-E and 1-E. In addition to the classical viroporin inhibitors amantadine, rimantadine and HMA, ivermectin and three of its derivatives, namely ivermectin aglycon, nemadectin and milbemycin were tested (Fig. 1A). A sequence alignment between SARS-CoV and all CoV-2 variants shows high conservation between E proteins and identifies the four out of 76 residues that are exchanged between 1-E and 2-E (Fig. 1B). The protein exhibits a pentameric structure with a central pore (Fig. 1C), and the C-terminus—containing the four exchanges—of each subunit pointing away from the channel pore. Recombinant expression of 1-E has been shown before [32]. 2-E protein was expressed in HEK293 cells and Western blot analysis confirmed the expression of the target protein (Fig. 1D). A membrane-directing signal peptide had been added to promote transport of E protein channels to the cell surface. A Western blot of 2-E with or without fused signal peptide (Fig. 1D) showed immune signals corresponding to the 2-E protein as monomer, dimers and possible trimers. Controls were the p7-1a viroporin of hepatitis C virus, also showing oligomers, and the SARS-CoV-2 ORF3a [40]. Due to the instability of the E-protein, membrane preparation was carried out in the presence of protease inhibitors, and prior to loading, samples were heated only briefly to prevent degradation (see methods). This treatment preserved the E-protein signal, however, oligomers were not fully separated. Even then, prominent degradation bands could be observed (Fig. 1D). The 2-E-SP conjugate showed only one band in the expected monomer region, but no band for the E-protein conjugated to the signal peptide, indicating efficient cleavage of the signal peptide. Overall the experiment indicates successful recombinant expression of the E-protein in HEK 293 cells. Cell surface expression was verified by a Dot blot assay (Fig. 1E), where cells were first transfected with 2-E constructs, and live cells treated with primary (anti-myc) and secondary antibodies before cell lysis and direct spotting onto a blot membrane. In this way, only protein on the outer cell surface was accessible to the antibody. Dot blots showed an immune signal on the cell surface, verifying plasma membrane expression, and indicated that cell surface expression was highest for 2-E protein containing the signal peptide (Fig. 1E).

**Fig. 2:**
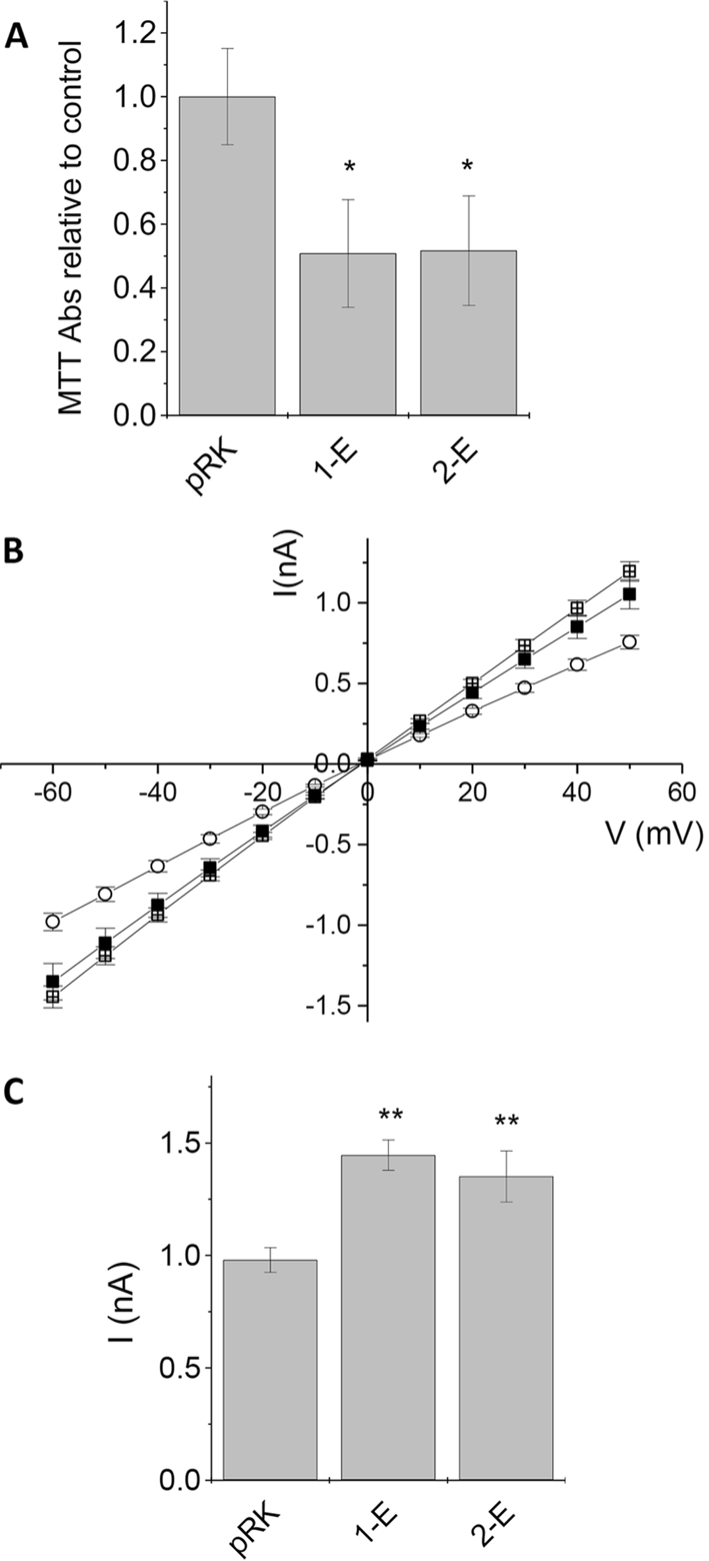
**A** Comparison of SARS-CoV 1-E and 2-E analysed by MTT assay. Absorbances at 595 nm were normalized to mock control (pRK vector). **B** Electrophysiological measurements on transfected HEK293 cells. 1-E (crossed square) and 2-E (solid square) transfected cells were compared to Mock (empty pRK vector, open circles) transfected control cells. Error bars are standard deviation (SD); number of cells were 16 (pRK) and 14 (1-E, 2-E). **C** Summary of electro physiological data: average currents at − 60 mV are shown ± standard deviation, significance (one-way ANOVA) is indicated, *: p < 0.05, **: p < 0.01

*Activity of E protein* We compared the activity of E protein from SARS-CoV to its new variant from SARS-CoV-2 in a cell viability assay and patch-clamp electrophysiological measurements. Control for both experiments were cells transfected with empty vector that were otherwise treated in the same way as the test experiment. In the cell viability assay, presence of active E protein weakened and eventually damaged cells, so reduced cell viability indicates E protein activity. Indeed, observed viabilities of HEK293 cells expressing E protein or a control construct were significantly different with the E protein causing a reduction of the viability of transfected cells (Fig. 2). The activities of both E proteins were almost identical, both reduced the fraction of viable cells to ~ half the level of the control (Viability levels Control: 1.00 ± 0.16, 1-E = 0.51 ± 0.17, 2-E = 0.52 ± 0.17, Fig. 2A).

We then compared the channel activity of both E proteins using whole-cell patch clamp electrophysiology where transmembrane channel activity, i.e., ion flux across the membrane is measured directly. We analysed current–voltage relationships in a range from − 60 to + 50 mV. Control cells showed background (leakage) currents of − 0.98 ± 0.05 nA at − 60 mV. Expression of E protein should induce larger currents due to its channel activity. As expected, currents after viroporin expression were significantly increased compared to control (Fig. 2B). When comparing both E proteins under identical conditions, 2-E-mediated currents were slightly lower, however, this difference was not significant (1-E = 1.45 ± 0.07 nA, 2-E = 1.35 ± 0.11 nA, p = 0.28).

**Fig. 3:**
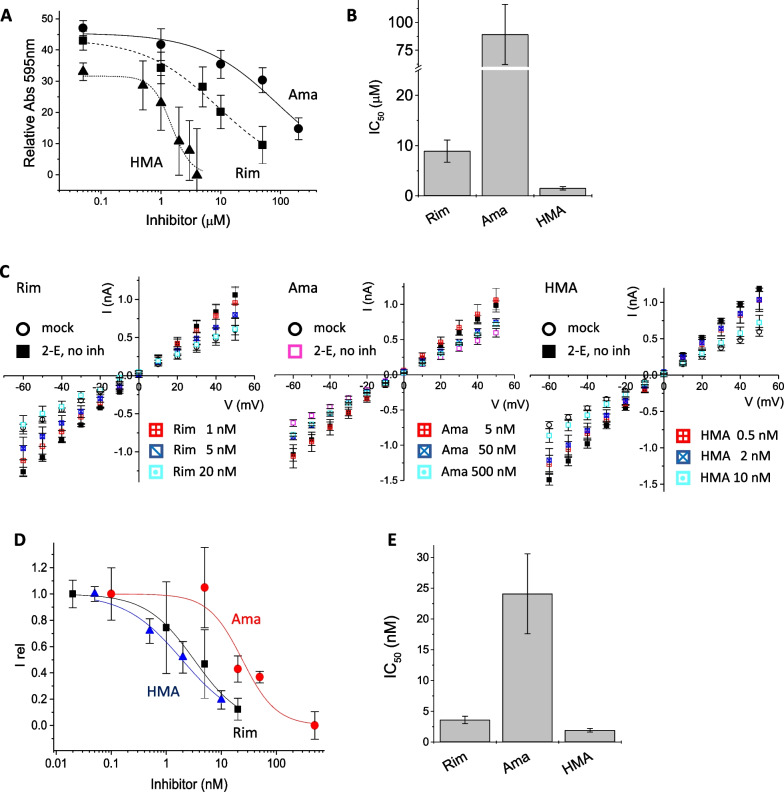
Inhibition of 2-E protein by classical viroporin inhibitors rimantadine (Rim), amantadine (Ama) and Hexamethylene amiloride (HMA). **A** Cell viability assay. Relative absorbance was normalized to control (mock transfection). **B** Summary of IC50 data. **C** Current-voltage relation of recombinantly expressed 2-E in HEK293 cells. Transmembrane voltage was stepped in 10 mV intervals in the range from − 60 to + 50 mV and associated currents recorded. Controls were mock (empty pRK vector) transfected HEK293 cells. Expression of 2-E resulted in increased currents that were reduced in the presence of inhibitor. Panels from left to right represent Rim, Ama and HMA. Concentrations are indicated in the Figure. **D** Current-voltage relationships for Rim, Ama and HMA were converted into an IC_50_ curve at −60mV and normalized between Irel = 0 (mock-transfected) and Irel = 1 (SARS-CoV-2 E protein without inhibitor); Irel = relative current. **E** Summary of IC50 data of Rim, Ama and HMA from patch-clamp electrophysiology

*Inhibition by rimantadine, amantadine and HMA* We investigated the activity of classical viroporin inhibitors on 2-E protein and compared it to that of the 1-E protein, which had been published earlier [32]. In the cell viability assay, 2-E protein expression in HEK293 cells and inhibition with rimantadine, amantadine and HMA showed IC_50_ values of 8.9 ± 2.2 µM, 89 ± 27 µM and 1.5 ± 0.3 µM, respectively (Fig. 3A, B). These activities were comparable to the inhibition data from published 1-E protein (IC_50_ of Rim = 10.9 ± 3.3 µM, Ama = 77 ± 13 µM, HMA = 2.6 ± 0.4 µM) [32]. While amantadine and rimantadine were not cytotoxic in the tested concentration range, HMA showed cytotoxic effects at concentrations > 10 µM, as had been reported before [29, 41]. Electrophysiological measurements on 2-E expressing HEK293 cells (Fig. 3C) revealed the same pattern of activity for all classical inhibitors with HMA being the most active, next rimantadine, and amantadine being the least active (Rim IC_50_ = 3.6 ± 0.6 nM, Ama IC_50_ = 24.1 ± 6.5 nM, HMA IC_50_ = 1.9 ± 0.3 nM, Fig. 3 D, E). It is noted that patch-clamp electrophysiology was performed directly on the cells where the E protein was present in the outer plasma membrane due to the membrane-directing signal sequence. This experimental configuration allows a direct interaction of the E protein channel and the inhibitor. IC_50_ values of the inhibitors established from patch-clamp experiments are thus much smaller than those obtained from cellular assays where the inhibitor first has to enter cells before binding to its target. When comparing patch-clamp inhibition data of 2-E to those of 1-E, we observed the same pattern of inhibitory potency (HMA > rimantadine > amantadine) for both channels, while there is a small difference in the extent of inhibition, with reduced activity for 2-E compared to 1-E (factor of ~ 2).

**Fig. 4:**
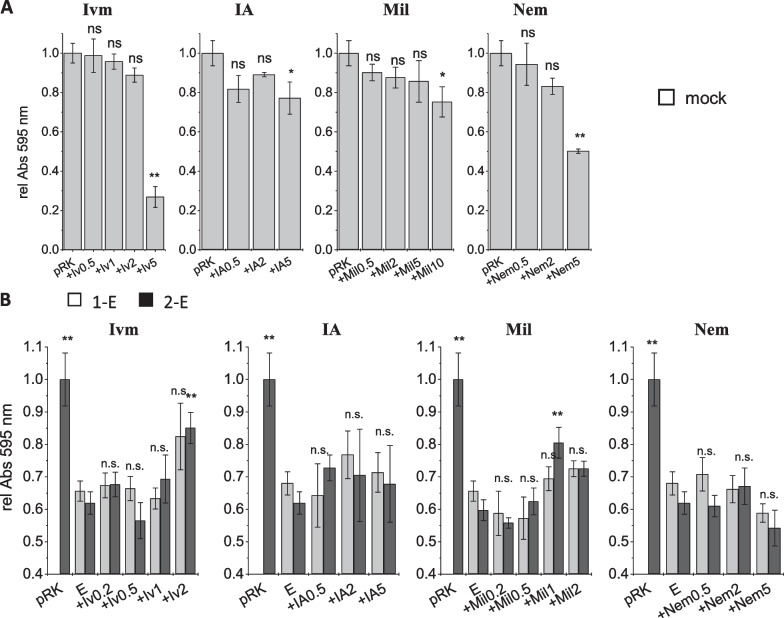
Cell viability test for inhibition of SARS-CoV and CoV-2 E protein by ivermectin (Ivm) and derivatives. **A** Cytotoxicity test on control cells (mock-transfected). Abs_595_ indicates cell viability, statistical significances relative to control (no inhibitor) values (on-way ANOVA) are indicated. Ivermectin derivatives were cytotoxic in concentrations ≥ 5 µM, except milbemycin, where the onset of toxicity started at ~10 µM. **B** Cell viability assays after recobinant 1-E (grey colums) and 2-E (black columns) expression in HEK293 cells. Inhibitors were applyed after transfection. Ivermectin (Ivm) and milbemycin (Mil) were tested in a range of 0.2 to 2 µM while ivermectin agylcon (IAP and nemadectin (Nem) were tested at 0.5 to 5 µM. Control was pRK mock-transfected cells. Cell viability of viroporin-transfected cells was normalized to the viability of control (mock-transfected) cells exposed to the same concentration of inhibitor. Statistical significance (one-way ANOVA) relative to the viability values of 1-E or 2-E without inhibitor is indicated, ns: non significant, *: p < 0.05, **: p < 0.01

**Fig. 5:**
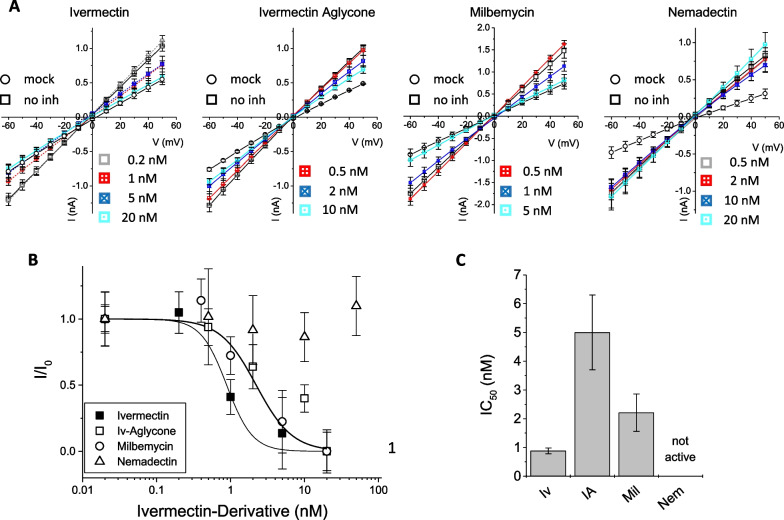
Patch-clamp electrophysiology tests for inhibition of SARS-CoV-2 E protein by ivermectin and derivatives. **A** Current-voltage relation of recombinantly expressed mock controls and 2-E protein. Applied voltages were between −60 to +50 mV. Control currents were taken from mock (pRK vector) transfected HEK293 cells. Panels from left to right show ivermectin (Iv), ivermectin aglycone (IA), milbemycin (Mil) and nemadectin (Nem). Concentrations are indicated in the graphs. **B** Concentration-response curves: currents at -60 mV were corrected for leak current (mock-transfected cells); the corrected currents observed for 2-E in presence of inhibitor were divided by the corrected currents in absence of inhibitor. Symbols are indicated in the graph. **C** Summary of the IC_50_ values of ivermectin derivatives against recombinant 2-E

*Inhibition by ivermectin and derivatives* As a control, we studied the effect of ivermectin, ivermectin aglycon, milbemycin and nemadectin on mock-transfected HEK293 cells in a cell viability assay. All ivermectin derivatives showed notable cytotoxicity. The onset of the cell toxicity varied between the experiments and was most pronounced for nemadectin where cell damage was observed already at 2 µM (Fig. 4A). The inhibitor concentration ranges (0.2–2 µM for ivermectin and milbemycin; 0.5–5 µM for ivermectin aglycon and nemadectin) were chosen based on simultaneously performed electrophysiological data showing higher activities for ivermectin and milbemycin than for ivermectin aglycon and nemadectin. For all ivermectin derivatives, inhibition of the 2-E protein in the cell viability assay (Fig. 4B) was less distinct than observed in former assays for classical inhibitors or flavonoids [32]. Only ivermectin showed inhibiting effects at the highest concentration tested (2 µM). One reason for the reduced apparent inhibitory potency of ivermectin derivatives might be the toxicity of the compounds themselves which overlays the inhibiting effect. Also, ivermectin and the derivatives are membrane-associated and may not reach the intracellular destination at the ER-Golgi membrane easily. In electrophysiological measurements, toxicity aspects are irrelevant since all drug concentrations are much smaller and hence non-toxic, while the second potential limitation in the cell viability assay—ivermectin not reaching its intracellular destination—doesn’t apply here, since viroporins are exposed on the outer plasma membrane. E protein-mediated currents were measured in voltage ramps between − 60 and + 50 mV recorded in the absence and presence of increasing concentrations of inhibitor (Fig. 5A). Complete inhibition resulted in currents that were reduced to the magnitude of mock-transfected HEK293 cells. We plotted relative currents against inhibitor concentration and determined IC_50_ values using a modified form of the Hill equation ${I}_{inh}={I}_{ctr}\bullet \frac{{IC}_{50}^{n}}{{IC}_{50}^{n}+[{inh]}^{n}}$, where I_inh_ is the current in presence of inhibitor, I_ctr_ is the current in absence of inhibitor, [inh] is the concentration of inhibitor, and n is the Hill coefficient for inhibition (Fig. 5B). Our measurements showed inhibition of E protein-mediated currents by all ivermectin derivatives except nemadectin. IC_50_ values were: IC_50_ (ivermectin) = 0.88 ± 0.1 nM, IC_50_ (ivermectin aglycon) = 5.0 ± 1.3 nM and IC_50_ (milbemycin) = 2.21 ± 0.65 µM. We did not observe inhibition of the 2-E protein by nemadectin at any of the tested concentrations. In summary, we can note that ivermectin and some of its derivates inhibit the E protein in the direct application (electrophysiology) while the inhibition in the indirect cellular assay (cell viability assay) was largely reduced for ivermectin and not measurable for its derivatives.

## Discussion

The anthelminthic avermectin was discovered in 1975 as an isolate from naturally occurring bacteria in soil found near Tokyo, Japan [33]. Despite intense search, the Japanese microorganisms remain the only source of avermectin ever found. Ivermectin, a safe and more effective avermectin derivative was initially introduced as a commercial product for Animal Health in 1981. It is effective against a wide range of parasites, including gastrointestinal roundworms, lungworms, mites, lice and hornflies [33]. In human health, ivermectin became most famous for its activity against a variety of internal infections caused by nematodes, including Onchocerciasis (river blindness), Ascariasis, Lymphatic filariasis, Gnathostomiasis, Trichuriasis and others [33, 42]. The anthelmintic drug ivermectin works by inhibiting neuronal activity and muscular contractility in arthropods and nematodes. The classical target of its antiparasitic action is believed to be an ivermectin-sensitive glutamate-gated chloride channel receptor (GluClR) that exists in a number of invertebrates [34]. GluClRs belong to the pentameric Cys-loop receptor family of ligand-gated ion channels and are found exclusively in invertebrates. Ivermectin in higher concentrations also activates vertebrate Cys-loop receptors, including the excitatory nicotinic and the inhibitory GABA type-A and glycine receptors [34]. Ivermectin is known to bind to the inhibitory glycine receptor at a specific site, acting as a full agonist [43, 44] while it acts as positive allosteric effector of GABA_A_ receptor and alpha7 neuronal nicotinic acetylcholine receptor [45, 46]. While EC_50_ values of ivermectin on glycine receptor are in the µM range, its potency as agonist of GluClR in invertebrates is 1000-fold higher with EC_50_ values in the low nM range [47]. This is the reason why ivermectin is highly effective against nematodes but poses no health risk to humans in low concentrations. It was indeed hoped that the E protein or orf3a from SARS-CoVs could be a potential target for ivermectin.

Here, we investigated the activity of ivermectin on the E protein of SARS-CoV-2 using a combination of a cell viability assay and whole-cell patch-clamp electrophysiological recordings. Western blot and Dot blot assays confirmed expression of the E protein on the outer membrane of HEK293 cells and efficient cleavage of the membrane-directing signal peptide. Expression of trace amounts of recombinantly expressed E protein on the plasma membrane of host cells has been reported [48], which could be enhanced by addition of the signal peptide. The cell viability assay had been introduced for p7 channels [29] and SARS-CoV E protein [32] and is a complementation of direct channel activity measurements of patch-clamp electrophysiology.

First, we compared the activity of the E proteins of SARS-CoV (1-E) and SARS-CoV-2 (2-E) using both assays, finding the same level of cell toxicity (no significant difference) of recombinant 1-E and 2-E protein expressed in HEK293 cells. Consistently, patch-clamp recordings confirmed similar levels of activity for 1-E and 2-E proteins after recombinant expression in HEK293 cells. Thus, exchange of four amino acid residues between 1-E and 2-E (Fig. 1B) did not seem to affect ion channel activity. Next, we compared the sensitivity of the both SARS-CoV E proteins against classical channel blockers, which had already published for 1-E [32]. In the cell viability assay, IC_50_ values of amantadine, rimantadine, and HMA were similar against 1-E and 2-E. In electrophysiological recordings, we observed a factor of 2 between both variants, while the order of sensitivity to the three inhibitors stayed the same. Most potent inhibitor was HMA, then rimantadine and least active was amantadine. The observed differences between the E protein variants 1-E and 2-E were most probably due to expression-specific differences, particularly since sequence of inhibitory potency and general channel activity were unchanged between both E protein variants.

Finally, we investigated the effect of ivermectin and three derivatives on the 2-E protein. Recently, ivermectin was shown to exhibit a 5000-fold reduction in SARS-CoV-2 viral RNA in vitro in Vero-h/SLAM cells [49]. Several mechanisms were proposed by which ivermectin might inhibit SARS-CoV-2 induced COVID-19. Molecular docking approaches suggest that inhibition by ivermectin of RNA-dependent RNA polymerase, which is required for viral replication, inhibits COVID-19 [50], while another study found that ivermectin targets importin-α/β1 heterodimer resulting in abolition of nuclear transport of SARS-CoV-2 [51]. To our knowledge, data on the 2-E protein being a potential binding partner for ivermectin have not been published until now. Indeed, numerous in vivo studies were published over the last two years, showing considerably varying results, a discrepancy that can be rationalized when the combined effect of the antiviral and cytotoxic activities of ivermectin are considered. Studies included the use of ivermectin in prophylaxis, against mild to moderate COVID-19 cases, and with patients needing intensive care. While some studies consider ivermectin to be the first agent effective for both prevention of COVID-19 and for treatment of all phases including outpatient treatment of the early symptomatic phase [37, 52–54]; other studies find that ivermectin has no effect at all [55, 56]. Several studies found protecting effects of ivermectin in the treatment of patients, however, the differences were not statistically significant or low certainty of evidence was reported [39, 57]. Despite the unclear clinical situation and warnings against its use, ivermectin had been promoted for treatment of COVID-19 in several countries.

Our study concentrated on in vitro investigation to find out if ivermectin acts on the E channel protein of SARS-CoV-2. The E channel is expressed intracellularly in the endoplasmic reticulum-Golgi intermediate compartment (ERGIC) [23]. Therefore, in the cell viability assay, any drug has to enter the transfected HEK293 cells, only then its inhibiting action can reduce the cell damage due to E protein activity. Ivermectin is hydrophobic and membrane associated, in case of glycine receptor its binding pocket is located inside the extracellular transmembrane region [58]. Ivermectin might not enter the cells in a concentration that is high enough to inhibit the E protein effectively. In our experiments available concentrations of the drug were limited since ivermectin and its derivatives were cytotoxic at concentrations around 2–10 µM, i.e. in the concentration range required for E protein inhibition. Ivermectin toxicity at therapeutic doses was observed before on chick primary fibroblast cells [36]. In patch-clamp electrophysiology, we used a plasmid that included a signal sequence to direct the E protein channel to the outer membrane. Here, the viroporin was exposed to inhibitors in the extracellular buffer and we were able to study the activity of applied inhibitors directly. This was the reason for lower IC_50_ values as compared to the indirect cell viability assay for the classical inhibitors. For ivermectin and some of its derivatives, we found an IC_50_ value similar to that of rimantadine (IC_50_ = 3.6 ± 0.6 nM). Using current–voltage relationships measured in presence and absence of inhibitor, we found ivermectin having the highest activity (IC_50_ = 0.88 ± 0.1 nM), milbemycin was slightly less active (IC_50_ = 2.21 ± 0.65 nM), followed by ivermectin aglycon (IC_50_ = 5.0 ± 1.3 nM); nemadectin was inactive in our measurements. Our in vitro electrophysiology data show that ivermectin indeed acts as an inhibitor on the E channel with a potency similar to that of rimantadine. This activity could not be shown in cell viability assays, probably due to inefficient drug delivery into the intracellular compartments of HEK293 cells and the cell toxicity of ivermectin and its derivatives. Our data is in agreement with literature findings showing that ivermectin has some beneficial effects in treatment of SARS-CoV-2 infected patients. The discrepancies that were observed could be explained by the inefficiency of drug delivery. Most studies described modest effects for COVID-19 infected patients that were on the borderline of statistical significance. When prophylaxis was investigated, the positive effect of ivermectin treatment was more pronounced. These findings agree with ivermectin having an inhibiting effect on the virus—against the E protein or other proteins—but with a low efficiency. If the virus load is high already at a time where patients observe COVID-19 symptoms, ivermectin treatment has no effect. In the case of prophylactic application, ivermectin might be able to inhibit the spreading of the virus, albeit only at concentrations where there is already a risk of cytotoxic side effects.

## Conclusions

This study shows that the E protein of SARS-CoV and SARS-CoV-2 is directly targeted by antiviral drugs. Ivermectin and some of its derivatives may be potent inhibitors of the E protein channel, yet their cytotoxicity may render them unsuitable for therapeutic application. Thus, activity against the viral target protein as well as compromising side effects have to be evaluated carefully. Viroporins, such as the E- and 3a proteins of SARS-CoVs remain promising and relevant targets in the search for novel antiviral drugs.

## Data Availability

The datasets used and/or analysed during the current study are available from the corresponding author on reasonable request.
